# Post-thaw CD34+ cell recovery likely degraded under extreme graft platelet concentrations

**DOI:** 10.1038/s41409-024-02409-w

**Published:** 2024-09-16

**Authors:** Gustavo C. Duarte, Leandro Ladvanszky, Gavin Atkinson, Michelle Burns, Dona Madola, Deepak Sadani, Marcus Yan-Fischer, Hemant Patel, Jacynthe Tremblay, Andrew Butler, Niranjan Rathod, Wen-Hua Wei

**Affiliations:** 1 New Zealand Blood Service, 15 Lester Lane, Christchurch, New Zealand; 2 New Zealand Blood Service, 71 Great South Road, Auckland, New Zealand; 3 New Zealand Blood Service, 183 Pembroke Street, Hamilton, New Zealand; 4 Haematology Department, Christchurch Hospital, 2 Riccarton Avenue, Christchurch, New Zealand; 5 Haematology Department, Waikato Hospital, 183 Pembroke Street, Hamilton, New Zealand; 6 Centre for Biostatistics, Division of Population Health, Health Services Research and Primary Care, The University of Manchester, Manchester, M13 9PL UK

**Keywords:** Stem-cell research, Stem-cell therapies

## Abstract

Impaired post-thaw CD34 cell (postCD34) viability in autologous haematopoietic stem cell transplant (ASCT) could indicate delayed engraftment where multiple factors might complicate the relationship. Despite of a couple of unconfirmed reports of a negative correlation of platelet concentration with postCD34 viability, how platelets might be involved in the relationship is largely unknown. Therefore, this question was addressed in this retrospective study of 82 ASCT patients with a total of 150 collections of peripheral blood stem cells in New Zealand. A significant negative correction between platelet concentration and postCD34 recovery (r = −0.18, *p* = 0.028) was observed overall, but upon further analysis only confirmed in the subset with graft platelets 1500–2000 ×10^9^/L. Importantly, the postCD34 recovery was clearly reduced in the subgroups with either the lowest or the highest platelet concentration. The lowest subgroup was enriched with collections from patients with Hodgkin or non-Hodgkin lymphoma, whereas the highest subgroup from patients with multiple myeloma, both with clearly male preponderance. We hypothesized that graft platelet concentrations probably indicated CD34 cell state (e.g. cell cycle and cell adhesion highly related to platelet functions) that sustained when platelet concentrations were within a niche range but went out of kilter otherwise.

## Highlights

- The first comprehensive analysis of the roles of platelets in post-thaw CD34 recovery.
- Platelet counts varied widely across peripheral blood stem cell collections.
- Post-thaw CD34 recovery reduced when graft platelet counts were very low or high.
- Patient gender, age and cancer type all influenced the reduction of CD34 recovery.

## Introduction

Autologous haematopoietic stem cell transplant (ASCT) is an essential tool in the treatment of haematological malignancies, such as lymphoma and multiple myeloma [1]. ASCT is often performed from mobilized peripheral blood stem cells (PBSC) collected by apheresis [1, 2]. The traditional strategy is to cryopreserve and store the cells under controlled temperature so the patient can be submitted to the conditioning chemotherapy and subsequently receive the cells after reinfusion [1, 2]. Despite this approach being used for decades, the cryopreservation and thawing process inevitably trigger stem cell loss [3]. Depending on the number of cells initially available before the cryopreservation, the final product infused might have a suboptimal number of cells increasing risks of delayed engraftment, infection, and blood transfusion requirement [4].

Many variables can be associated with cell loss during the cryopreservation and thaw processes, including pre-cryopreservation counts of viable CD34-positive (CD34) cells and white cells [3, 5]. Additional variables such as previous therapies, underlying disease, age, and gender have been explored but without a conclusive answer [5]. These known variables jointly could explain only a small proportion of the variation in cell loss, implicating that most patients who presented substantial cell loss did not have an identifiable cause [5]. Moreover, how these variables work together during the processes remains largely unknown.

Recently, Valentini et al. [6] reported an inverse correlation between post-thaw CD34 cell (postCD34) viability and graft platelet concentration in a retrospective study of 146 apheresis procedures and cryopreservation. The authors found that the group with lower postCD34 viability had a higher graft platelet concentration than that in the counterpart group with higher postCD34 viability (*p* < 0.001), and postulated that platelets could impact stem cell viability via the release of inflammatory mediators or by the formation of clumps [6]. Nevertheless, this finding is yet to be replicated and the current guidelines do not make recommendations suggesting that platelet concentration should be assessed [7–9]. Furthermore, Akbar et al. did observe a negative association of platelet count with postCD34 viability in a simple logistic regression that was marginally significant but disappeared after correction for covariates in a multiple logistic regression analysis [10].

Therefore, this study was conducted to closely examine the effects of graft platelet concentration on CD34 cells of patients submitted to stem cell collection and cryopreservation.

## Material and methods

Medical records from adult patients (≥18 years old) submitted to peripheral blood stem cell collection, cryopreservation, and post-thaw recovery assessment for treatment of lymphoma and multiple myeloma at the Christchurch and Waikato Hospitals, New Zealand, from January 2020 to October 2023, were retrospectively reviewed. Patient demographic data, including gender and age at stem cell harvesting, mobilization regimen, stem cell yield, and white cell and platelet concentration of the graft were analysed. A total of 82 patients with a total of 150 collections were included in the study.

Peripheral blood stem cells (i.e. PBSC) were mobilized with granulocyte colony-stimulating factor (G-CSF) with or without chemotherapy and plerixafor according to disease and patient conditions. Leukapheresis was performed using automated system apheresis equipment (Spectra Optia, Terumo BCT, Lakewood, CO, USA) with ACD-A as the anticoagulant agent. Products were processed immediately after harvest or stored overnight at 4 °C and processed the following morning. The white cell count (WCC) and platelet count were performed before the product was submitted to plasma reduction, using a Sysmex cell counter (Sysmex XS-1000i or Sysmex XN-1000, Sysmex Corporation, Kobe, Japan). After centrifugation and plasma volume reduction using a manual plasma extractor, peripheral blood stem cells were diluted 1:2 with DMSO 10% and autologous plasma. All PBSC products were frozen in a controlled-rate freezer and stored in vapour phase nitrogen tanks below −150 °C. Reference samples were prepared and cryopreserved with the main unit. Viable CD34 enumeration before processing and three to seven days after being cryopreserved were assessed by flow cytometry according to the ISHAGE protocol. The viable CD34 recovery (vCD34) was calculated as the percentage of the viable postCD34 compared to the pre-processing viable CD34.

**Fig. 1:**
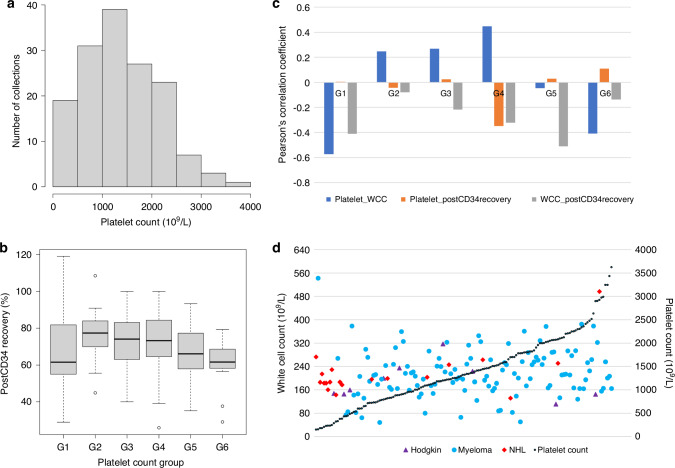
Extreme platelet counts associated with reduced post-thaw viable CD34 cell recovery, cancer type and/or cell state. **a** Histogram of platelet counts in 150 apheresis collections; **b** boxplots of viable post-thaw CD34 cell (postCD34) recovery by platelet count group; **c** patterns of Pearson’s correlation coefficients by platelet count group, where Platelet_WCC: correlation between platelet and white cell counts, Platelet_postCD34recovery: correlation between platelet count and post-thaw CD34 cell viability, WCC_postCD34recovery: white cell count and post-thaw CD34 cell recovery; **d** co-distributions of white cell counts along with platelet counts sorted in ascending order, where platelet counts in black dotted line by the second y axis to the right, corresponding white cell counts per collection in either purple triangulars representing Hodgkin, or blue dots representing Myeloma, or red diamonds represent NHL.

Statistical analyses were performed using R (v4.1.1) and R packages *MASS* and *stats*. Pearson’s correlations were computed using the *rcorr()* function with p-values calculated based on two-tailed t tests. To facilitate the data analysis, we derived an additional variable of platelet Group by grouping graft platelet concentrations according to their distributions in the PBSC collections as showed in Fig. 1a. Considering post-thaw viable CD34 recovery per collection as the outcome variable, a three-step model selection approach was taken to perform multivariable linear regression analyses using the *lm()* function and the *stepAIC()* function: (1) fitting the NULL model where all variables of interest without any interactions were fitted in a linear regression model; (2) fitting the FULL model where all variables of interest and their all possible interactions were fitted in a linear regression model; (3) the results of the NULL and the FULL models were fitted into the *stepAIC()* function with the direction parameter set as ‘both’ to perform a stepwise selection of the best model explaining the most phenotypic variance. The resultant best model concerns only covariates (i.e., variables and/or their interactions) with significant effects in the regression and was further analysed using the *anova()* function to quantify variance explained by each covariate and to examine significance using the built-in F test.

All methods were performed in accordance with the relevant guidelines and regulations. Written informed consent was available from all patients. According to the Standard Operating Procedures for the Health and Disability Ethics Committees of New Zealand, as a minimal risk, retrospective, observational study, with non-identifiable data, it did not require ethical committee approval. (https://ethics.health.govt.nz/).

## Results

Table 1 summarized 82 ASCT patients with a total of 150 collections of PBSCs performed at Christchurch Hospital and Waikato Hospital, New Zealand, from January 2020 to October 2023, of which 81.3% collected for Multiple Myeloma (Myeloma), 7.3% for non-Hodgkin lymphoma (NHL), and remaining 2.7% for Hodgkin lymphoma (Hodgkin). Patients had a male preponderance in Myeloma and NHL, with a median age of 60 years and on average 1.8 collections. Isolated G-CSF was the mobilizing agent in the majority of cases (61%), with chemo-mobilization being more frequent in the lymphoma groups. A high utilization of plerixafor was observed in the Hodgkin group (56%).

**Table 1:** Summary information of ASCT cases and apheresis collections^a^.

	All	Hodgkin	Myeloma	NHL
Total patients	82	4	67	11
Male (%)	52 (63.4%)	2 (50%)	43 (64.2%)	7 (63.6%)
Median age (year)	60.0	45.5.0	60.0	58.0
Collection	150	9	122	19
Mobilization regimen				
G-CSF	92 (61%)	1(11%)	86 (70%)	5 (26%)
G-CSF + Chemotherapy	30 (20%)	3 (33%)^b^	17 (14%)^c^	10 (53%)^d^
G-CSF + Plerixafor	28 (19%)	5 (56%)	19 (16%)	4 (21%)
preCD34 (10^6^/kg)	3.4 ± 2.9	3.6 ± 3.8	3.6 ± 2.9	2.2 ± 1.5
WCC (10^9^/L)	214.5 ± 79.3	187.1 ± 63.6	216.3 ± 80.7	216.2 ± 77.9
Platelet (10^9^/L)	1398.8 ± 763.0	1156.0 ± 855.9	1514.0 ± 705.7	773.9 ± 783.8
postCD34 (10^6^/kg)	2.4 ± 2.4	2.4 ± 3.0	2.5 ± 2.4	1.3 ± 0.8
postCD34 recovery	70.3% ± 16.5%	67.3% ± 18.9%	71.2% ± 16.1%	65.9% ± 18.3%

Standard deviation for each count and viability included after the ± symbol.

*WCC* white cell count, *Platelet* platelet count, postCD34 post-thaw CD34 cell count, *postCD34 recovery* post-thaw CD34 cell recovery calculated as percentage of postCD34 out of preCD34.

^a^preCD34 – CD34 cell count before processing/cryopreservation.

^b^Gemcitabine 2 g/m^2^, Cisplatin 75 mg/m^2^ and Dexamethasone 160 mg.

^c^Cyclophosphamide 2 g/m^2^.

^d^Etoposide 300 mg/m^2^, Carboplatin up to 800 mg, Ifosfamide 5 g/m^2^ with or without Rituximab 375 mg/m^2^.

The average postCD34 recovery was 70.3% which differed across cancer types with the highest of 71.2% in Myeloma. As illustrated in Supplementary Fig. S1, both WCC and platelet counts showed very good concordance in the pre-collection complete blood count and in the graft, with a Pearson’s correlation coefficient of 0.89 (*p* = 6.2e−10) and 0.87 (*p* = 2.4e−09) respectively. Interestingly, the average platelet counts had certain concordance with the postCD34 recovery by cancer type, the highest was also in Myeloma, followed by that in Hodgkin and then NHL (Table 1).

Indeed, from the data described above we observed a significant negative correlation between platelet count and postCD34 recovery (r = −0.18, *p* = 0.0278), in addition to a known negative correlation between WCC and postCD34 recovery (r = −0.30, *p* = 0.0002) from our previous study [5] and elsewhere. WCC was nearly normally distributed (Supplementary Fig. S2) and was weakly correlated with platelet count positively (r = 0.15, *p* = 0.06). However, an in-depth subgroup analysis, as indicated by the histogram of platelet counts (Fig. 1a), revealed striking variations across plate count groups (Table 2, Fig. 1b) leading to new insights into postCD34 recovery.

**Table 2:** Post-thaw CD34 recovery by platelet count groups^a^.

	G1	G2	G3	G4	G5	G6
Platelet range (10^9^/L)	<500	[500,1000)	[1000, 1500)	[1500, 2000)	[2000, 2500)	≥2500
Collection	18	32	39	26	24	11
Mobilization regimen						
G-CSF	4 (22%)	19 (59%)	25 (64%)	19 (73%)	16 (67%)	9 (82%)
G-CSF + Chemotherapy	14 (78%)	6 (19%)	3 (8%)	3 (12%)	3 (12%)	1 (9%)
G-CSF + Plerixafor	0 (0%)	7 (22%)	11 (28%)	4 (15%)	5 (21%)	1 (9%)
PostCD34 recovery (%)						
Median	61.6	77.4	74.1	73.2	66.1	61.7
Mean ± SD	67.8 ± 22.8	75.9 ± 12.4	71.7 ± 15.3	71.2 ± 17.6	66.4 ± 15.5	60.1 ± 14.9
Collection (<60%)	8	3	10	6	7	3
Proportion (<60%)	44.4%	9.4%	25.6%	23.1%	29.2%	27.3%

^a^*Platelet range* a range of platelet counts in 10^9^/L where ‘[’ representing greater than or equal to (≥) and ‘)’ representing less than (<), *postCD34 recovery* post-thaw viable CD34 cell recovery calculated as percentage of viable postCD34 out of the viable preCD34, *SD* standard deviation, *collection or proportion (<60%)* number of collections with postCD34 recovery less than 60% or percent of such collections out of the total collections in each group.

The G1 and G6 groups with extreme platelet counts (<500 ×10^9^/L and ≥2500 ×10^9^/L respectively) clearly stood out from the remaining groups with reduced postCD34 recovery (Table 2 and Fig. 1b). The G1 group also had the highest variability in postCD34 recovery as indicated in the boxplot and the highest standard deviation of 22.8% reflected by the fact that the median was lower than the mean by 6% (Table 2). In contrast to the correlations calculated from the full data above, a strong negative correlation of platelet count with postCD34 recovery was observed only in the G4 group, whereas the correlation between platelet count and WCC was strongly negative in both the G1 and G6 groups (Fig. 1c). Plotting the WCC of each collection against their corresponding platelet counts in ascending order, we found that most collections in the G1 group were from NHL and Hodgkin patients, in contrast to the G6 group most were from Myeloma patients (Fig. 1d, Supplementary Table S1). The proportion of patients receiving chemotherapy and G-CSF as the mobilization regimen was higher in group one, accounting for 78% of this group and almost 47% of all patients undergoing this regimen (Table 2). Interestingly, WCC varied within a relatively small range except for two outliers one each in the G1 and G6 groups respectively.

A multivariate regression analysis of postCD34 recovery confirmed that there were significant statistical interactions between diagnosis (or cancer type) and platelet group which explained 9.1% of the total variance (Supplementary Table S2). The regression model also detected significant effects carried by WCC and platelet group, as well as significant interactions of the platelet group with age and gender, and between gender and diagnosis (Supplementary Table S2). The interaction between gender and platelet group appeared to be clinically important indicating that the pattern of postCD34 recovery changes with the platelet group in male patients was different from that in female patients, particularly in the G1 and G6 groups (Supplementary Fig. S3) where the male preponderance was the highest (about 3 times more than females, Supplementary Table S1).

For the 28 collections with G-CSF + plerixafor, the correlation of WCC with CD34 recovery rate was −0.32 (*p* = 0.098). In contrast, of 122 collections that not using plerixafor, the correlation was −0.31 (*p* = 0.00057). Given a small data, our results did not support the hypotheses of plerixafor usage as an independent factor association with lower CD34 recovery (Supplementary Table S2).

## Discussion

This paper examines the potential roles of graft platelet count in relation to postCD34 recovery and issues in apheresis stem cell collection and processing. Instead of finding a replication of the reported negative correlation of platelet count with postCD34 viability, we discovered that postCD34 recovery was clearly reduced when platelet count went either very high (i.e. the G6 group) or very low (i.e. the G1 group). It was further discovered that the G1 group was enriched with collections from NHL and Hodgkin patients often submitted to more intensive mobilization strategies and the G6 group was enriched with collections from Myeloma patients, and both groups had elevated male preponderance.

These results indicated that postCD34 recovery reduction could be associated with cancer types and gender, which was confirmed in the multivariate regression analysis. Considering WCC varied within a relatively narrow range (Fig. 1d and Supplementary Fig. S2), graft platelet counts probably indicated an underlying CD34 cell state that sustained when platelet counts were within a niche range but went out of kilter when platelet counts went extremes. Unfortunately, we don’t have information available about the cancer, such as the staging and previous chemotherapies, to better dissect this variable.

The observation of reduced postCD34 recovery in G1 reflected the fact that NHL and Hodgkin patients tended to have much lower platelet counts in the graft than Myeloma patients as showed in Table 1. NHL and Hodgkin’s patients were often mobilized with chemotherapy before apheresis collection, which could result in low platelet counts in the blood stream. The strong correlation between the blood stream platelet count and the graft platelet count (Supplementary Fig. S1) supports this explanation. Furthermore, of a total of 30 “Chemo + G-CSF”, 13 were non-Myeloma: 12 in Group1, 1 NHL-B with platelet count of 720 ×10^9^/L in Group 2. It can be argued that the expected myelotoxicity of the chemotherapy used for lymphomas is higher than the drugs used for myeloma, explaining the fact from 17 Myeloma, only 3 were in Group1. Hence, the observation coincided with the delayed engraftment platelet recoveries in NHL patients in our previous study using the same protocol [5]. While it is of interest to see how well the delayed platelet recovery might be replicated in NHL patients of the current study cohort, it is intriguing why most but not all NHL patients had rather low platelet concentrations. A similar question can be asked why rather high platelet concentrations presented only in some Myeloma patients. Much of the answer would probably come from oncology and pathophysiology studies but the clues could be highly informative to apheresis collection and cell processing for these patients.

It is unclear how the platelet content of the graft could be associated with stem cell survival. One possibility is that increased platelet content and, therefore, degranulation during the cryopreservation process would lead to an inflammatory response and cell loss, as seen in G6. On the other hand, in an early review of PBSC transplant, delayed platelet recovery in heavily pre-treated patients undergone intensive chemotherapy was discussed along with CD34 cell state in terms of the disrupted cell cycle by mobilization and endogenous factors such as endogenous cytokines and/or the haematopoietic microenvironment [11]. Further studies confirmed the impact of cell cycle and micro-environment [12, 13]. Furthermore, the composition of CD34 cell subpopulations appeared to be significantly different across sources of stem cell collections and thus could be used to predict engraftment kinetics and immune reconstitution in recipients [14, 15]. It is plausible to hypothesize that the same causes promoting different graft platelet content could also be associated with different inflammatory response patterns, stem cell repertoire and functional capabilities. Hence, if the platelets have a direct role determining cell viability or act as a surrogate marker of the underlying “cell state”, it is still undetermined, and further investigation must take place.

This study highlighted the need for comprehensive analysis accompanied by careful dissection of clinical observations when studying complicated clinical problems. As reported at the beginning of the Results section, using only Pearson’s correlation analysis of all data would lead to a wrong conclusion that platelet count was significantly negatively correlated with postCD34 recovery, which in fact was observed only in the G4 group (Fig. 1c). The wrong conclusion could even be considered as a replication of Valentini et al. [6] if not careful. Indeed, taking a close look at the means (variation range) and distribution of raw platelet count reported in Table 1 and Figure 3 of Valentini et al. [6] respectively, one could easily tell that the higher mean of platelet counts was driven largely by two outliers with extremely high platelet counts. Another example is the characterisation of the G1 group where median and mean (Table 2) and recovery (Fig. 1b) together made a nearly complete view and thus enabled in-depth analysis to derive the clinical insights into this odd group. It is important to bear in mind that the timing of the platelet assessment in the graft can be different among studies. In this study, we assessed the platelet content before the processing and addition of the cryopreservation agents and hence, dilution of the cell concentration. If the assessment was done immediately before cryopreservation, the figures would be different.

The current report also showed that modelling statistical interactions in complex clinical problems was beneficial in revealing valuable new insights into the clinical complex that otherwise would have gone missing. The identified statistical interactions not only explained the extra variance of the complex problem but also helped with the interpretation of the observed data patterns (Fig. 1d) in a clinically meaningful way (Supplementary Fig. S3). For example, considering interactions between gender, cancer type and platelet group together could facilitate deriving clinical hypothesis to be tested to better understanding of the G1 and G6 groups. Nonetheless, cares should be taken when modelling and interpreting statistical interactions in small data that are vulnerable to risk of over-parametrization. In any case, replication in independent data is highly recommended.

Our study has some limitations. Firstly, the sample size is relatively small because it is not the current practice to monitor the platelet concentration of the PBSC units. Secondly, the retrospective nature is associated with inevitable biases. Thirdly, engraftment outcomes are yet to become available to assess the impact of reduced postCD34 recovery. Moreover, our study defined the viable post-thaw CD34 recovery as the end-point and Valentini, as the post-thaw CD34 viability. The postCD34 recovery is based on the comparison between the viable CD34 cell enumeration in the pre-processing and the post-cryopreservation samples. On the other hand, CD34 viability is assessed independently in each sample as a percentage of the total CD34 cells (viable and non-viable). Therefore, although recovery and viability are informing the underlying quality of the graft, and they tend to be generally concordant, they cannot be interpreted as synonyms.

In conclusion, extreme graft platelet counts indicate a risk of reduced postCD34 recovery. To validate these findings, further studies enroling a larger sample and with comprehensive data about the patients should be performed.

## Supplementary information

Supplementary Figures legends

Table S2

Table S1

Figure S1

Figure S2

Figure S3

## Data Availability

The de-identified data that support the findings of this study are available from the corresponding author upon reasonable request.
