# Supplementary material for: Post-thaw CD34+ cell recovery likely degraded under extreme graft platelet concentrations
Source: Bone Marrow Transplant. 2024 Sep 16;59(12):1704–9. doi: 10.1038/s41409-024-02409-w (PMC11611725; doi:10.1038/s41409-024-02409-w)
Supplement: Supplementary file 1 — Supplementary Figures legends [file 41409_2024_2409_MOESM1_ESM.docx]

Figure S1: Concordance of white cell count and platelet count in the pre-collection Complete Blood count (blood stream) and in the graft.

Figure S2: Histogram of white cell counts in 150 apheresis collections

Figure S3: Graphic display of interaction between gender and platelet group
