# Supplementary material for: Post-thaw CD34+ cell recovery likely degraded under extreme graft platelet concentrations
Source: Bone Marrow Transplant. 2024 Sep 16;59(12):1704–9. doi: 10.1038/s41409-024-02409-w (PMC11611725; doi:10.1038/s41409-024-02409-w)
Supplement: Supplementary file 2 — Table S2 [file 41409_2024_2409_MOESM2_ESM.docx]

Table S2: Analysis of variance of the resultant multivariate regression model*

Analysis of variance of the resultant multivariate regression model*

|  | Df | Sum_Sq | F_value | Pr(>F) | %variance |
| --- | --- | --- | --- | --- | --- |
| Gender | 1 | 44.8 | 0.2021 | 0.65380 | 0.1% |
| Age | 1 | 63.0 | 0.2837 | 0.59519 | 0.2% |
| Diagnosis | 2 | 747.1 | 1.6833 | 0.18971 | 1.8% |
| plGroup | 5 | 2915.4 | 2.6277 | 0.02672 | 7.2% |
| WCC | 1 | 3446.8 | 15.5334 | 1.3e-04 | 8.5% |
| mobilization | 2 | 387.4 | 0.8728 | 0.42016 | 1.0% |
| Diagnosis : mobilization | 4 | 2842.7 | 3.2028 | 0.01513 | 7.0% |
| Gender : Diagnosis | 1 | 924.7 | 4.1673 | 0.04320 | 2.3% |
| Residuals | 132 | 29290.3 |  |  | 72.0% |

*: plGroup: platelet count group; WCC: white cell count; statistical interaction representing by ‘:’; Df: degrees of freedom; Sum_Sq: sum of squares; F_value: value of the F test; Pr(>F): p value of the F test; %variance: percentage of the total sum of squares (i.e. variance) explained
