# Supplementary material for: Post-thaw CD34+ cell recovery likely degraded under extreme graft platelet concentrations
Source: Bone Marrow Transplant. 2024 Sep 16;59(12):1704–9. doi: 10.1038/s41409-024-02409-w (PMC11611725; doi:10.1038/s41409-024-02409-w)
Supplement: Supplementary file 3 — Table S1 [file 41409_2024_2409_MOESM3_ESM.docx]

Table S1: Distribution of apheresis collections by platelet groups and by diagnosis or gender

Distribution of apheresis collections by platelet groups and by diagnosis or gender

|  | G1 | G2 | G3 | G4 | G5 | G6 |
| --- | --- | --- | --- | --- | --- | --- |
| Platelet range (10^9^/L) | <500 | [500,1000) | [1000, 1500) | [1500, 2000) | [2000, 2500) | >=2500 |
| Hodgkin | 3 | 2 | 2 | 0 | 1 | 1 |
| Myeloma | 4 | 28 | 34 | 25 | 22 | 9 |
| NHL | 11 | 2 | 3 | 1 | 1 | 1 |
| Female | 4 | 15 | 20 | 11 | 8 | 3 |
| Male | 14 | 17 | 19 | 15 | 16 | 8 |

*: Platelet range – a range of platelet counts in 10^9^/L where ‘[’ representing greater than or equal to (>=) and ‘)’ representing less than (<).
