# Supplementary figures and images for: Post-thaw CD34+ cell recovery likely degraded under extreme graft platelet concentrations
Source: Bone Marrow Transplant. 2024 Sep 16;59(12):1704–9. doi: 10.1038/s41409-024-02409-w (PMC11611725; doi:10.1038/s41409-024-02409-w)

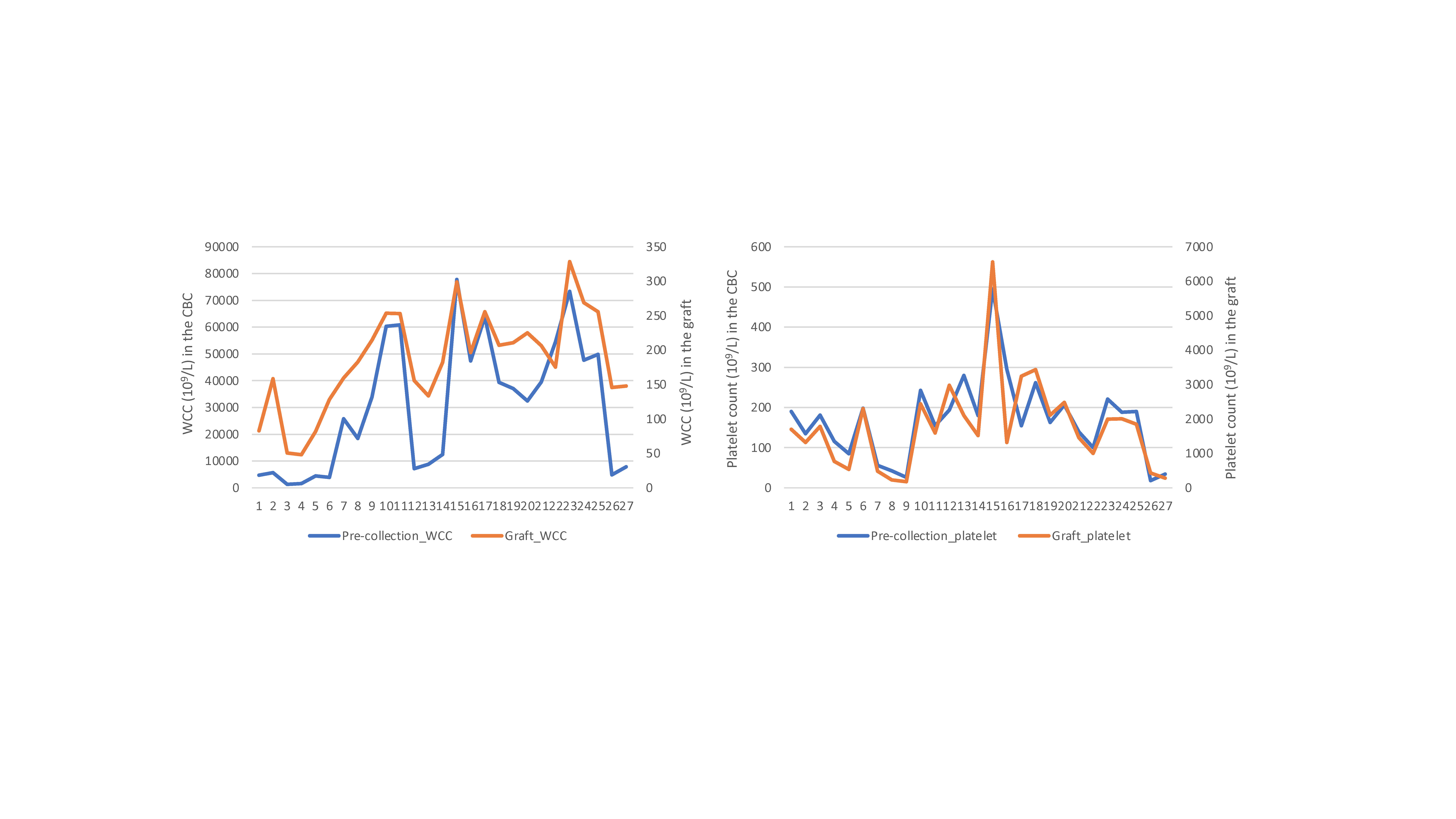

Supplement: Supplementary file 4 — Figure S1 [file 41409_2024_2409_MOESM4_ESM.jpg]

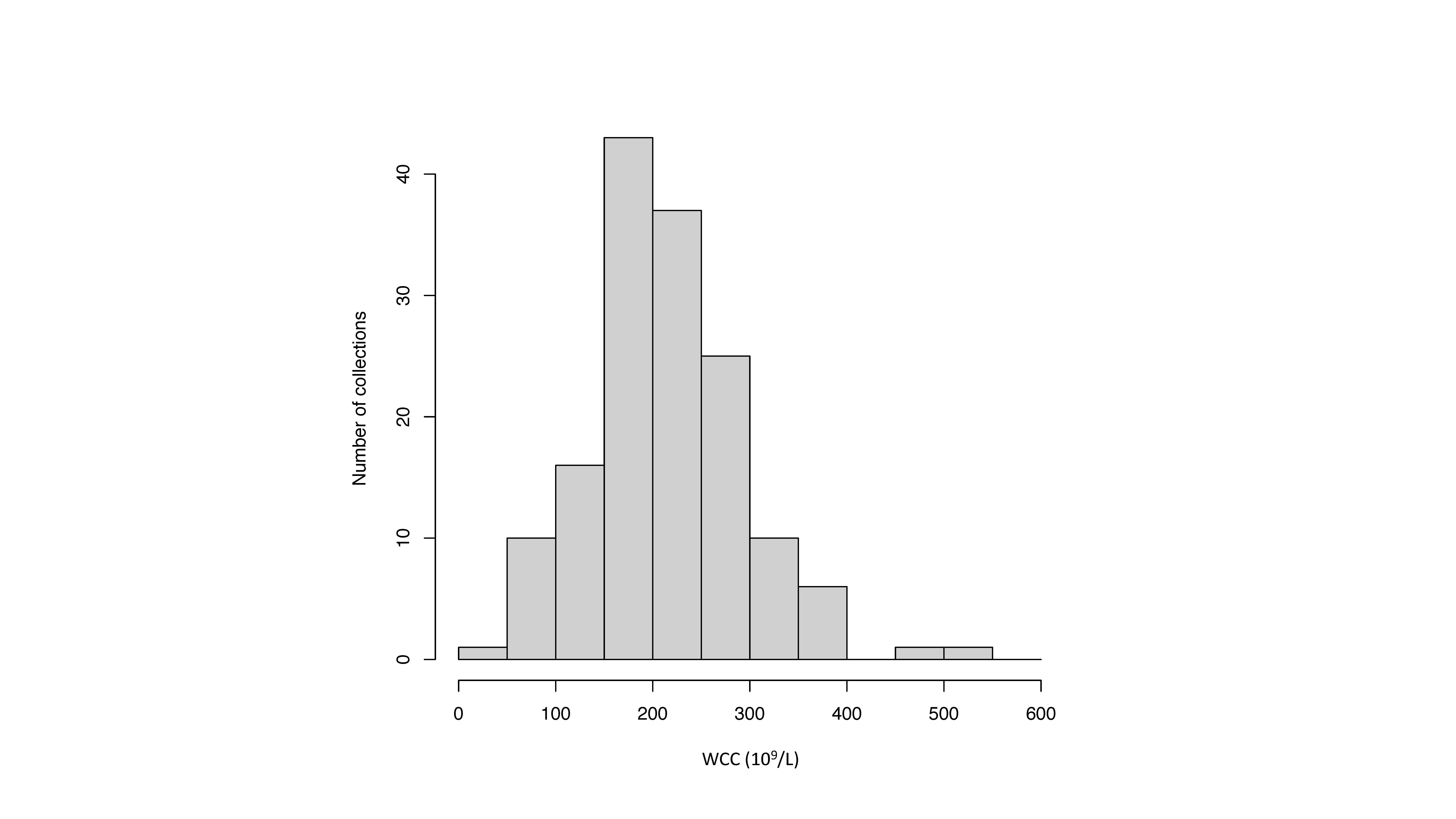

Supplement: Supplementary file 5 — Figure S2 [file 41409_2024_2409_MOESM5_ESM.jpg]

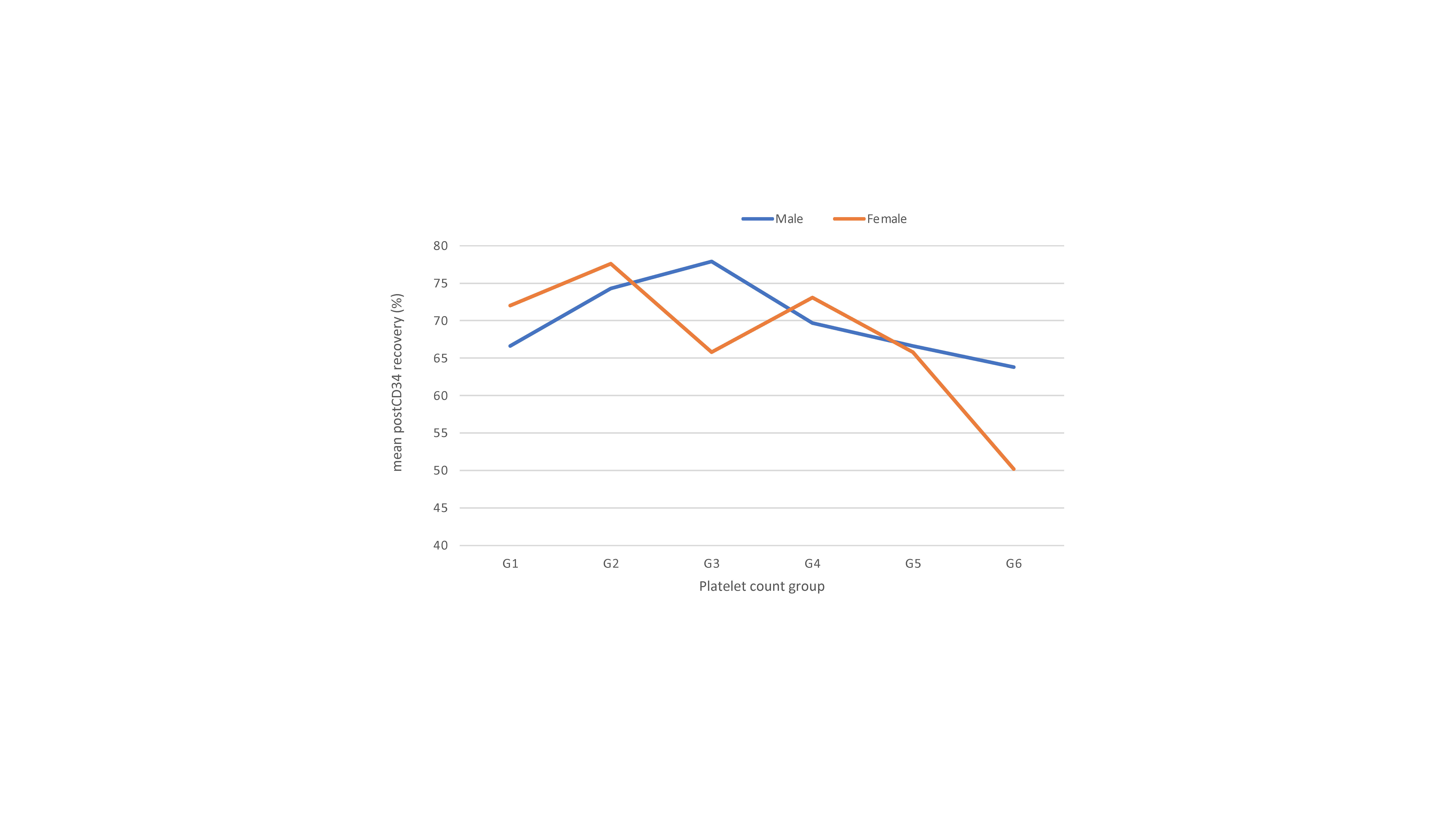

Supplement: Supplementary file 6 — Figure S3 [file 41409_2024_2409_MOESM6_ESM.jpg]
